# Toca-1 is suppressed by p53 to limit breast cancer cell invasion and tumor metastasis

**DOI:** 10.1186/s13058-014-0503-x

**Published:** 2014-12-30

**Authors:** Harish Chander, Colin D Brien, Peter Truesdell, Kathleen Watt, Jalna Meens, Colleen Schick, Doris Germain, Andrew WB Craig

**Affiliations:** 1 Department of Biomedical and Molecular Sciences, Queen’s University, Division of Cancer Biology and Genetics, Queen’s Cancer Research Institute, 10 Stuart Street, Kingston, K7L 3N6 Ontario Canada; 2 Centre for Genetic Diseases and Molecular Medicine, Central University of Punjab, Mansa Road, Bathinda, 151001 India; 3 Department of Medicine, Division of Hematology and Oncology, Tisch Cancer Institute, Mount Sinai School of Medicine, 1190 5th Avenue, New York, 10029 USA

## Abstract

**Introduction:**

Transducer of Cdc42-dependent actin assembly-1 (Toca-1) recruits actin regulatory proteins to invadopodia, and promotes breast tumor metastasis. Since metastatic breast tumors frequently harbor mutations in the tumor suppressor p53, we tested whether p53 regulates Toca-1 expression.

**Methods:**

Normal mammary epithelial cells (HBL-100, MCF10A) and breast cancer cell lines expressing wild-type (WT) p53 (DU4475, MTLn3) were treated with camptothecin or Nutlin-3 to stabilize p53 to test effects on Toca-1 mRNA and protein levels. Chromatin immunoprecipitation (ChIP) assays were performed to identify p53 binding site in Toca-1 gene. Stable silencing of p53 and Toca-1 were performed in MTLn3 cells to test effects on invadopodia and cell invasion *in vitro*, and tumor metastasis *in vivo*.

**Results:**

We observed that breast cancer cell lines with mutant p53 have high levels of Toca-1 compared to those with WT p53. Stabilization of WT p53 led to further reduction in Toca-1 mRNA and protein levels in normal breast epithelial cells and breast cancer cells. ChIP assays revealed p53 binding within intron 2 of *toca1*, and reduced histone acetylation within its promoter region upon p53 upregulation or activation. Stable silencing of WT p53 in MTLn3 cells led to increased extracellular matrix degradation and cell invasion compared to control cells. Interestingly, the combined silencing of p53 and Toca-1 led to a partial rescue of these effects of p53 silencing *in vitro* and reduced lung metastases in mice. In human breast tumors, Toca-1 levels were high in subtypes with frequent p53 mutations, and high Toca-1 transcript levels correlated with increased risk of relapse.

**Conclusions:**

Based on these findings, we conclude that loss of p53 tumor suppressor function in breast cancers leads to upregulation of Toca-1, and results in enhanced risk of developing metastatic disease.

**Electronic supplementary material:**

The online version of this article (doi:10.1186/s13058-014-0503-x) contains supplementary material, which is available to authorized users.

## Introduction

Metastasis is a complex process in which tumor cells acquire the ability to spread to other tissues via lymphatics or blood vessels. Invading cancer cells form filamentous actin (F-actin)-based membrane protrusions called invadopodia, whose extracellular matrix (ECM) degrading activity allows them to invade through basement membranes and migrate toward blood vessels [1]. Silencing of key components of invadopodia such as N-WASP or cortactin, leads to impaired tumor vascularization and reduced metastasis in breast cancer models [2],[3]. Invadopodia formation is driven by epidermal growth factor receptor (EGFR) and Src kinase activation that induce recruitment of actin regulatory proteins (Cdc42/Toca-1/N-WASP, cortactin) required for F-actin branching [4],[5]. Transducer of Cdc42-dependent actin assembly-1 (Toca-1, also known as FNBP1L) was first identified as an essential adaptor protein to allow Cdc42 to release N-WASP from an autoinhibited state and recruit Arp2/3 complex [6]. Toca-1 is a member of the Cdc42-interacting protein-4 (CIP4) subfamily of Fer/CIP4 homology-Bin/Amphiphysin/RVS (F-BAR) proteins. The N-terminal F-BAR domain of Toca-1 forms a crescent-shaped dimer that targets Toca-1 to areas of membrane curvature [7],[8]. The central PKN homology region-1 (HR1) domain binds Cdc42^GTP^, and the C-terminal SH3 domain binds several actin regulatory proteins, including N-WASP [6], dynamin [7], diaphanous-related formins [9], Abi1 [10], and cortactin [11].

Several recent studies have identified functions of Toca-1 in regulating filopodia formation and vesicular trafficking in neuroblastoma cells [12], EGFR trafficking to lysosomes [13], and EGFR-driven cell motility and invasion [14]. We recently identified Toca-1 as a component of invadopodia in breast cancer cells, and that silencing of Toca-1 led to reduced incidence of metastasis to the lung in mammary orthotopic xenograft models [11]. In this study, we also reported that Toca-1 expression levels are high in triple-negative breast cancer (TNBC) cell lines, which lack expression of estrogen receptor (ER)/progesterone receptor (PR)/human epidermal growth factor receptor 2 (HER2) receptors.

TNBC frequently harbor mutations in the tumor suppressor p53, resulting in both loss-of-function and gain-of-function effects on p53 pathways [15],[16]. In addition to loss of an appropriate DNA damage response, these cancers are also more invasive due to upregulation of proteins involved in epithelial-mesenchymal transition (EMT) and cell invasion [17]. In smooth muscle cells, p53 limits podosome formation and cell invasion via expression of microRNAs (miRNAs) that silence key podosome inducers [18], and upregulation of caldesmon, a negative regulator of actin polymerization [19]. Although similar pathways may control invadopodia in cancer cells, the role of p53 in regulating invadopodia has not been reported.

In this study, we show that Toca-1 upregulation in TNBCs is due, at least in part, to loss of repression by wild-type (WT) p53. We further demonstrate that p53 suppresses invadopodia, cell invasion and tumor metastasis in breast cancers with WT p53. Silencing of Toca-1 can partially rescue the effects of p53 silencing in these systems, consistent with its role in promoting breast cancer cell invasion and tumor metastasis. Profiling of Toca-1 levels in human breast tumors revealed high Toca-1 levels in HER2 and TNBC that frequently harbor p53 mutations, and high Toca-1 transcript levels were associated with increased risk of relapse in patients with TNBC.

## Methods

### Cell lines and cell culture

All of the cell lines used in this study were acquired from American Type Culture Collection (ATCC, Rockville, MD, USA), and were grown in their recommended media. Briefly, Dulbecco’s modified Eagle’s medium (DMEM) supplemented with 10% fetal bovine serum (FBS) was used for BT-20, MCF-7, SKBR3, MDA-MB 435, MDA-MB 468 and BT474 cells. RPMI 1640 supplemented with 10% FBS was used for HBL-100, ZR75-1, T47D and HCC-1500 cells. RPMI 1640 supplemented with 10% FBS, 1% 1 M HEPES, 1% sodium pyruvate, 1% D-glucose, 1% glutamine and 1% penicillin/streptomycin was used for DU4475, HCC-1187 and HCC-38 cells. MCF10A cells were grown in DMEM Nutrient Mixture F-12 HAM. HCC-2157 were grown in DMEM-F12 media plus supplements (10% FBS, 0.01 mg/mL transferrin, 0.02 mg/mL insulin, 25 nM sodium selenite, 50 nM hydrocortisone, 1 ng/mL EGF, 0.01 mM ethanolamine, 0.01 mM phosphorylethanolamine, 0.5% (w/v) bovine serum albumin (BSA), 0.5 mM sodium pyruvate, and 50 pM triiodothyronine).

### Transfections

All plasmid transfections were done using Xtreme Gene HP DNA (Roche, Basel, Switzerland) in serum-free media followed by replacement with complete media. Small interfering RNA (siRNA) against human p53 was from Santa Cruz Biotechnology (Dallas, TX, USA) (sc-29435). Hiperfect transfection reagent (Qiagen, Venlo, The Netherlands) was used to transfect siRNA.

### Induction of p53

MCF-10A and DU4475 cells were plated 24 hours before treatment with either Nutlin-3 (Sigma-Aldrich, St Louis, MO, USA) or camptothecin (CPT, Sigma-Aldrich, Oakville, ON, Canada) for 0 to 48 hours at doses indicated in figure legends. Cells were then harvested, pelleted, lysed in Nonidet-P40 (NP-40) lysis buffer (1% NP-40, 137 mM NaCl, 20 mM Tris HCl pH 8, 10% glycerol and 2 mM EDTA) and protein extract was collected. Immunoblot analysis was then performed using Toca-1, p21, p53 and β-actin antibodies.

### RNA isolation and quantitative reverse transcription-polymerase chain reaction (qRT-PCR)

Total RNA extraction was done using the RNeasy Plus Mini Kit (Qiagen) according to the manufacturer’s instructions. cDNA synthesis was performed with random hexamer primers and Superscript II reverse transcriptase (Invitrogen, Waltham, MA, USA). qRT-PCR was performed on cDNA using human Toca-1 primers (for 5′ CAAACCAGGAAGTCCGTGGGCC 3′; Rev 5′ ATGTCACACATGGCACAAAGGTGC 3′) and GAPDH primers (for 5′ GCCTTCCGTGTCCCCACTGC 3′; Rev 5′ CAATGCCAGCCCCAGCGTCA 3′) and SYBR Green JumpStart Taq ReadyMix kit (Sigma-Aldrich; 58°C annealing, 40 cycles, BioRad iCycler (BioRad Laboratories, Hercules, CA, USA). Transcript levels were analyzed using the 2^-ΔΔCT^ method [20]. Toca-1 mRNA levels were normalized to glyceraldehyde-3-phosphate dehydrogenase (GAPDH) mRNA levels.

### Immunoblot analysis and antibodies

Non-commercial antibody mouse anti-Toca-1 was kindly provided by Giorgio Scita (IFOM, Italy) as previously described [13]. Commercial sources of antibodies included anti-p53 (DO-1; human-specific; sc-126), anti-p21 (sc-56335), anti-β-Actin (sc-47778), and immunoglobulin G (IgG) controls (sc-2762, sc-2763) were all from Santa Cruz Biotechnology. For MTLn3 cell studies, mouse anti-rat p53 antibody was used (1C12; Cell Signaling Technology, Beverly, MA. USA). Secondary antibodies included horseradish peroxidase (HRP)-conjugated anti-mouse or anti-rabbit IgGs (GE Healthcare, Little Chalfont, UK). Immunoblot analyses were performed as described previously [11].

### Chromatin immunoprecipitation (ChIP) assays

HBL-100 and DU4475 cells were treated with or without CPT (10 μM) for 24 hours prior to harvesting. For assays with ectopic WT p53 expression, HCC1806 cells were transfected with WT p53 for 24 to 48 h prior to harvesting. ChIP assays were performed as described previously [21]. Briefly, cells were crosslinked with 1% formaldehyde (Sigma-Aldrich) followed by lysis of cells in RIPA buffer (50 mM Tris pH 8.0, 5 mM EDTA, 150 mM NaCl, 1% NP-40, 0.5% DOC, 0.1% SDS) and sonication (10 sec on/off cycle, 20 pulses performed four times; Thermo Fisher Scientific (Waltham, MA, USA, Sonic Dismembrator Model 500). DNA-protein complexes were immunoprecipitated using antibody (DO-1) against p53, acetylated histone H3 (H3K14ac) antibody (#06-599, EMD Millipore, Billerica, MA, USA), or a control IgG (Santa Cruz Biotechnology). After reversal of crosslinks and purification (Qia-quick PCR Purification Kit, Qiagen), DNA was subjected to PCR using the primers from the predicted binding sites of p53 within intron 2 of *toca1* (SA Biosciences, Frederick, MD, USA (GPH1006475 (-) (01A)), or p21 promoter primers. The acetyl-histone H3 (H3K14Ac) precipitates were subjected to qPCR using primers from proximal promoter region of Toca-1. PCR reactions were performed using 5 μL of the DNA from the immunoprecipitations or the 2 μL of DNA from the input as template. PCR cycling conditions were as follows: 94°C for 3 min; then 35 cycles of 94°C for 45 s, 58°C for 45 s, and 72°C for 45 s; followed by 10 min at 72°C as final extension.

### Stable silencing of p53 and Toca-1

For silencing of rat p53 in MTLn3 cells, an LMP retroviral vector expressing a p53 short hairpin RNA (shRNA) was previously described [19]. The system for lentiviral delivery of Toca-1 shRNA was also described previously [11]. Briefly, MTLn3 cells were incubated with retroviral or lentiviral particles individually or combined prior to selection with puromycin (2 μg/ml) and cell pools were passaged several times prior to testing for p53 and Toca-1 silencing. To test for effects of p53 and Toca-1 silencing on cell viability, cells (5,000) were seeded in 96-well plates in triplicate for 18 hours, and analyzed following addition of AlamarBlue™ for 5 hours using a plate-reading spectrophotometer to measure absorbance at 570 nm. Cell growth assays were conducted by plating 50,000 cells from each group in 60 mm plates on day 0, followed by total viable (trypan blue-negative) cell counts on days 2 and 3 using a hemocytometer.

### Extracellular matrix degradation assays

MTLn3 control, sh-p53 and sh-p53/sh-Toca-1 cells were seeded on gelatin-coated glass coverslips (15,000 cells) containing a layer of thin gelatin, prepared as described previously [4]. After incubation for 16 h, cells were fixed, permeabilized and stained with Alexa Fluor-488 Phalloidin (Life Technologies Inc., Burlington, ON, Canada). Epifluorescence microscopy was performed and the areas of ECM digestion beneath the cells that were stained with Phalloidin were quantified as digestion area per cell using Image Pro Plus software (Media Cybernetics, Bethesda, MD, USA). At least 100 cells were quantified for each condition and cell line.

### Transwell invasion assays

Invasion assays were performed as described previously [11]. Briefly, transwell inserts (8 μm pore) were coated with 100 μl of ice-cold Matrigel (1:5 dilution in DMEM with 0.5% FBS) for invasion, or coated with 0.1% (w/v) gelatin for migration assays, and incubated at 37°C for 30 min. A total of 50,000 cells were added in the upper chamber, which was placed in a 24-well plate containing serum as a chemoattractant. After overnight incubation, the filters were fixed in 4% paraformaldehyde, cells and Matrigel on the top of the filter were removed and the cells attached to the underside of the filter were stained with DAPI. Epifluorescence microscopy was performed to detect nuclei of cells that migrated to the underside of the filters. Scoring of invading cells from four separate fields was performed for triplicate filters using the Image Pro Plus software (Media Cybernetics).

### Mammary orthotopic tumor xenograft assays

All tumor xenograft assays were performed using Rag2^−/−^:IL-2Rγc^−/−^ mice (BALB/c background) as previously described [11],[22]. Animals were housed in a specific pathogen-free facility (Queen’s University Animal Care Services), with ventilated cages and sterilized food and water supply. All procedures with mice were approved by the Queen’s University Animal Care Committee. MTLn3 control, sh-p53 and sh-p53/sh-Toca-1 cells were grown to 70 to 85% confluence before trypsinization and counting. For xenograft assays, 0.5 × 10^6^ cells were injected into the right thoracic mammary fat pads in a volume of 50 μl of 50% Matrigel using a hypodermic syringe. At end points of 4 weeks mice were sacrificed and primary tumor mass recorded. Several tissues were removed for detection of metastases, which were primarily observed in the lungs. The primary tumors and lungs from each mouse were used for histological analysis. Samples were fixed in formalin and embedded in paraffin, and 5 μm sections were stained with hematoxylin and eosin (H&E). H&E-stained sections were scanned using the Aperio CS digital slide scanner (Queen’s Laboratory for Molecular Pathology) and analyzed with ImageScope software (Aperio Technologies, Vista, CA, USA). The number of metastases and overall lung area were measured in a blinded fashion.

### Immunohistochemistry (IHC)

Human breast cancer tissue microarrays (T086b, BR10010a, BR963 and BR953, US Biomax, Inc., Rockville, MD, USA) were stained using the Discovery XT Staining System (Ventana Medical Systems, Inc. Tucson, AZ, USA). Antigens were retrieved with an EDTA pH 8.0 solution and incubated with rabbit anti-p53 (DO-1) and Toca-1 antibody as used previously [11],[23]. Staining was visualized with DAB treatment and a hematoxylin counterstain. Microarrays were scanned using the Aperio CS digital slide scanner (Queen’s Laboratory for Molecular Pathology) and analyzed with ImageScope software (Aperio). Tumor-specific H-scores were calculated based on positive pixel intensity according to the formula: (% weak positive X 1) + (% positive X 2) + (% strong positive X 3). The p53 IHC scoring was grouped according to the extreme positive (EP), extreme negative (EN), and non-extreme (NE) categories that were recently shown to best relate to p53 mutation status and outcomes [24]. Toca-1 H-scores were reported according to p53 EP/EN or NE groups, and analyzed by Student’s *t* test.

## Results

### Toca-1 levels correlate with p53 status in breast cancer cell lines

**Figure 1:**
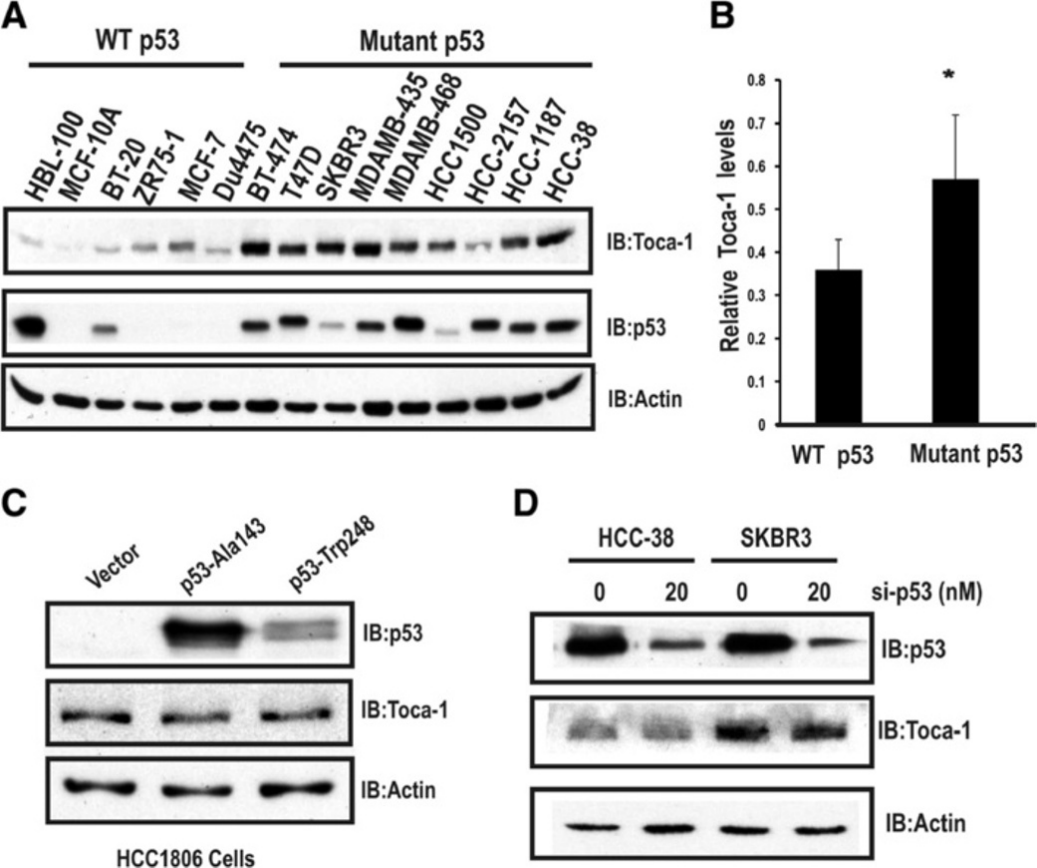
**Toca-1 expression correlates with p53 status in breast cancer cell lines. (A)** Lysates from the indicated normal-like breast epithelial and breast cancer cell lines were grouped according to either WT or mutant p53 and subjected to IB with Toca-1, p53 and β-Actin antibodies. **(B)** Densitometric analysis of Toca-1 protein levels relative to β-actin in WT p53 and mutant p53 cell lines (mean ± SD); ^*^depicts a significant difference based on paired Student’s *t* test (*P* <0.05)). **(C)** p53-null HCC1806 cells were transfected with mutant p53 (Ala-143, Trp-248) plasmids and the levels of Toca-1 and p53 were assessed by IB (actin served as a loading control). **(D)** siRNA mediated silencing of endogenous mutant p53 in HCC-38 and SKBR3 cell lines. Levels of Toca-1 and p53 were assessed by IB (actin served as a loading control). IB, immunoblot; siRNA, small interfering RNA; SD, standard deviation; Toca-1, transducer of Cdc42-mediated actin assembly; WT, wild-type.

We previously showed that Toca-1 levels were high in TNBC cell lines and a subset of invasive ductal carcinomas [11]. Since TNBCs have a high frequency of p53 mutations [15], we tested Toca-1 levels relative to p53 status in a panel of cell lines. This included two immortalized breast epithelial, normal-like cell lines (HBL-100 and MCF-10A), and a panel of human breast cancers with either WT p53 (BT-20, ZR75-1, MCF-7, DU4475), or mutant p53 (BT-474, T47D, SK-BR3, MDA-MB-435, HCC2157, MDA-MB-468, HCC-1500, HCC-1187, HCC-38). Lysates were subjected to immunoblot (IB) with Toca-1 and p53 antibodies. This analysis revealed high levels of Toca-1 in the vast majority of mutant p53 cell lines compared to WT p53 (Figure 1A). Similar results were observed for p53, with high levels of mutant p53 accumulating in these cells compared to WT p53 (Figure 1A; actin served as a loading control). In the case of HBL-100 cells, the high levels of WT p53 are likely due to presence of SV40 viral antigens [25] that cause p53 stabilization [26]. Using densitometry, the average levels of Toca-1 relative to β-actin was significantly higher in mutant p53 cell lines compared to WT p53 cell lines (Figure 1B). To test whether a similar correlation between p53 status and Toca-1 expression exists at the level of gene expression, we profiled Toca-1 mRNA levels across the same panel of cell lines by qRT-PCR. Toca-1 transcript levels were normalized to human GAPDH, and were found to be considerably higher in mutant p53 compared to WT p53 cancer cell lines (Figure S1 in Additional file 1). It is worth noting that the lowest levels of Toca-1 transcripts were observed in normal-like breast epithelial cell lines (Figure S1 in Additional file 1). Together, these results suggest that p53 status may dictate the levels of Toca-1 in normal cells and cancer cell lines.

Since mutant p53 confers both loss of WT p53 function, and gain-of-function effects on a large number of target genes [23],[27],[28], we asked whether Toca-1 expression was induced or dependent on mutant p53 expression. To test this, endogenous Toca-1 levels were analyzed upon ectopic expression of two frequently observed p53 mutants (p53-Ala143 and p53-Trp248) in p53-null HCC1806 cells. Surprisingly, the overexpression of either p53 mutant had no effect on Toca-1 levels (Figure 1C). To reinforce these results, siRNA-mediated knockdown (KD) of mutant p53 expression in HCC38 (TNBC) and SKBR3 (HER2) was performed. Although the levels of mutant p53 were greatly reduced in both cell lines, the levels of Toca-1 were unchanged (Figure 1D). These results suggest that overexpression of mutant p53 and gain-of-function effects are unlikely to explain the upregulation of Toca-1 in these breast cancer cell lines.

### WT p53 suppresses Toca-1 expression

**Figure 2:**
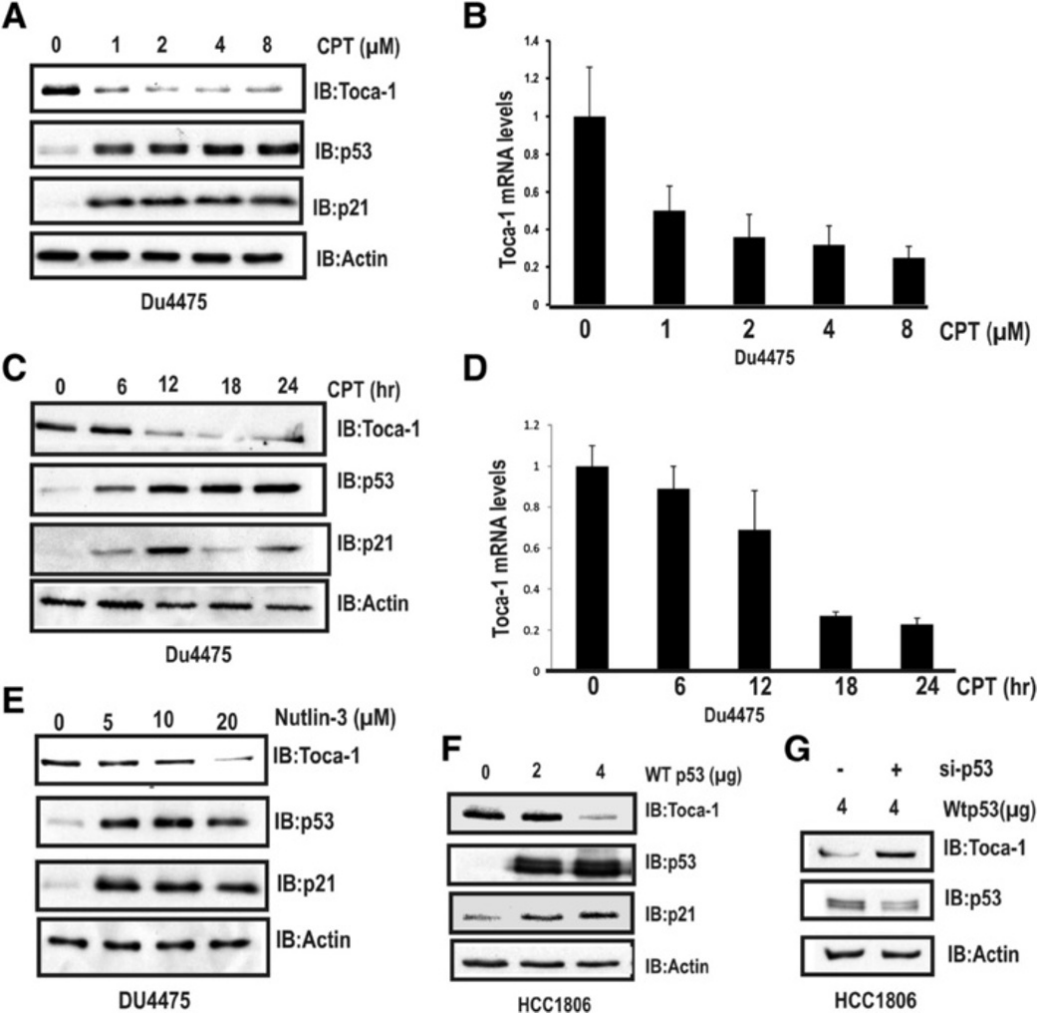
**WT p53 downregulates Toca-1. (A)** DU4475 cells were treated with indicated doses of CPT) for 24 h, and lysates subjected to IB with Toca-1, p53 and p21 antibodies. **(B)** Toca-1 mRNA levels were analyzed in DU4475 cells treated as above by qRT-PCR (2^-∆∆CT^ values for Toca-1 were normalized to GAPDH for each concentration of the drug, and graph depicts transcript levels relative to DMSO-treated cells (mean ± SD; triplicate samples)). **(C)** DU4475 cells were treated with CPT (4 μM) for the indicated times, and analyzed by IB as above. **(D)** DU4475 cells were treated with CPT (4 μM) for the indicated times were subjected to qRT-PCR analysis of Toca-1 mRNA levels as above. **(E)** DU4475 cells were treated with Nutlin-3 at indicated doses for 24 hours, and subjected to IB analysis as above. **(F)** p53-null HCC1806 cells were transfected with WT p53 expression plasmid (0 to 8 μg) for 24 to 48 hours, and levels of Toca-1, p53 and p21 were assessed by IB (β-actin served as a loading control). **(G)** HCC1806 cells were transfected with WT p53 plasmid, and after 24 hours with a siRNA against p53 prior to harvesting at 48 hours. Lysates were subjected to IB with Toca-1, p53 and β-actin antibodies. CPT, camptothecin; GAPDH, glyceraldehyde-3-phosphate dehydrogenase; IB, immunoblot; qRT-PCR, quantitative reverse transcription-polymerase chain reaction; SD, standard deviation; siRNA, small interfering RNA; Toca-1, transducer of Cdc42-mediated actin assembly; WT, wild-type.

We next tested whether Toca-1 expression could be repressed by physiological induction of endogenous p53 using camptothecin (CPT), a DNA-damaging agent that causes rapid stabilization of p53 [29]. Normal-like MCF10A cells were treated with increasing doses of CPT for 24 h (2 to 20 μM; DMSO served as vehicle control) prior to IB analysis of Toca-1, p53 and p21WAF1, which served as a positive control for p53 activation. As expected, CPT treatment led to increased p53 and p21 protein levels, and this coincided with reduced levels of Toca-1 at both the protein and mRNA levels (Figure S2 in Additional file 2). In DU4475 cells expressing low levels of WT p53, CPT treatment (1 to 8 μM) also led to a significant reduction in Toca-1 protein and mRNA levels along with the expected stabilization of p53 and upregulation of p21 (Figure 2A,B). We next analyzed the kinetics of p53 effects on Toca-1 expression by treating DU4475 cells with CPT (4 μM) for 0 to 24 h. We observed maximal levels of p53 and p21 proteins within 12 hours of CPT treatment, and a corresponding decrease in Toca-1 protein and mRNA levels at 18 and 24 h (Figure 2C,D). To further dissect this mechanism, we used Nutlin-3, a small molecule inhibitor of MDM2 that prevents targeting of p53 for proteasomal degradation. As with CPT, we observed a dose-dependent effect of Nutlin-3 on the stabilization of p53 and downregulation of Toca-1 in DU4475 cells (Figure 2E). These results are consistent with WT p53 stabilization, and not DNA damage *per se*, triggering downregulation of Toca-1 in breast cancers expressing WT p53.

To test whether WT p53 expression is sufficient for Toca-1 downregulation, p53-null HCC1806 cells were transfected with increasing amounts of a WT p53 expression plasmid. At the highest amount of plasmid corresponding to maximal p53 and p21 levels, we observed decreased levels of Toca-1 (Figure 2F). As an additional control, co-transfections of WT p53 plasmid with a p53 siRNA impaired the downregulation of Toca-1 in this model (Figure 2G). Thus, our data suggests that the loss-of-function effects of WT p53 are responsible for the elevated levels of Toca-1 in the breast cancer cell lines with p53 mutations.

### Toca-1 is a novel p53 target gene subjected to epigenetic regulation by p53

**Figure 3:**
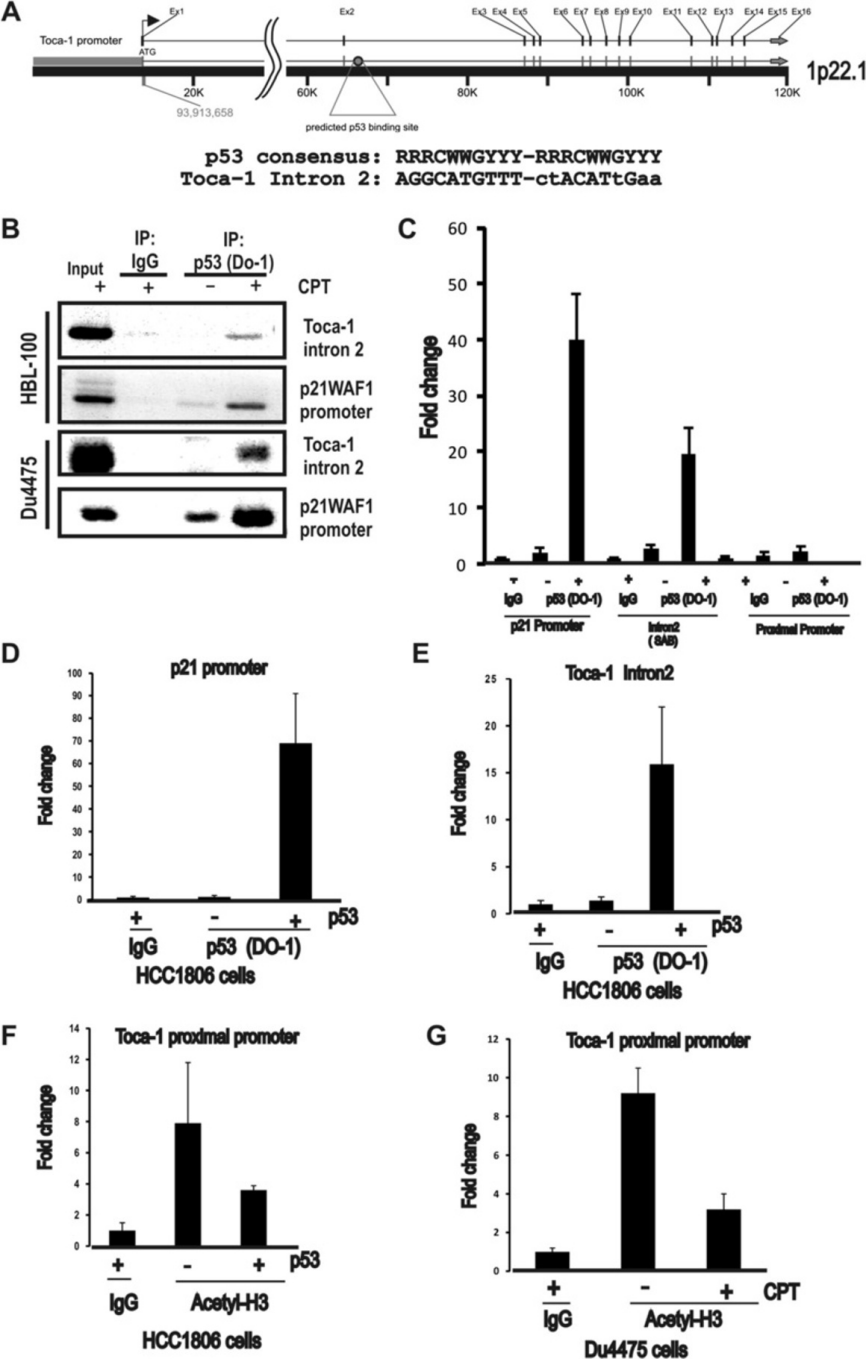
**p53 binding and altered chromatin marks at the** ***toca1*** **gene. (A)** *toca1* gene organization and a predicted p53 binding site within intron 2 is shown. Below, the consensus sequence for p53 binding is shown (R = A/G, W = A/T, and Y = C/T) relative to the predicted site, with lowercase letters denoting deviations from the consensus. **(B)** HBL-100 and DU4475 cells were treated with or without CPT (8 μM) to stabilize endogenous WT p53, and ChIP assays performed using control IgG or p53 antibody. Input material and ChIP assays were analyzed by PCR for intron 2 of toca1 and p21 promoter region as a positive control. Inverted images of representative ethidium bromide gels are shown. **(C)** DU4475 cells treated as above were subjected to ChIP assays and analyzed by qPCR. Graph depicts the fold change in p53 binding to the p21 promoter (positive control), or toca1 intron 2, or toca1 proximal promoter (mean ± SD; triplicate samples). **(D and E)** HCC1806 cells were transfected with or without WT p53 plasmid and ChIP assays performed with control IgG or p53 (DO-1) antibodies. qPCR analyses for the p53 binding site within p21 promoter region **(D)** and intron 2 of *toca1* **(E)** are shown (2^-∆∆CT^ values for p53 antibody were normalized to IgG; graph depicts the fold change in recovery with p53; mean ± SD; triplicate samples). **(F and G)** ChIP assays for acetylated histone H3 (H3K14Ac) were analyzed by qPCR for the proximal promoter region of Toca-1 in HCC1806 cells transfected with WT p53 **(F)** and CPT-treated DU4475 cells (**G**; 2^-∆∆CT^ values for p53 antibody were normalized to IgG; graph depicts the fold change in recovery with acetylated histone H3; mean ± SD; triplicate samples). ChIP, chromatin immunoprecipitation; CPT, camptothecin; IgG, immunoglobulin G; qPCR, quantitative polymerase chain reaction; SD, standard deviation; Toca-1, transducer of Cdc42-mediated actin assembly; WT, wild-type.

Since we observed similar suppression of Toca-1 protein and mRNA with WT p53 stabilization in these breast cancer models, we investigated whether Toca-1 is a previously unidentified p53 target gene. The Toca-1 gene (*toca1/fnbp1l*) includes 16 exons spanning 120 kb on chromosome 1 (1p22.1), and although no p53 response elements were predicted in the promoter region, a potential binding site was present in intron 2 of *toca1* (Figure 3A). This site included a perfect consensus for the first repeat, and an imperfect second repeat (Figure 3A). To test whether WT p53 binds this site, CPT-treated normal-like breast epithelial cells (HBL-100) and DU4475 breast cancer cells were subjected to ChIP assays using p53 antibody (DO-1) or a control IgG. PCR analyses revealed recovery of *toca1* intron 2 with p53 in CPT-treated cells compared to untreated or control IgG (Figure 3B). As expected, this also led to increased recovery of the p21 promoter region bound to p53 in both cell lines (Figure 3B). To quantify and extend on these results, we analyzed additional ChIP assays using qPCR and observed 40-fold and 20-fold enrichment in p53 binding to p21 promoter and *toca1* intron 2 with CPT treatment, respectively (Figure 3C). We also tested primers in the proximal promoter region of *toca1*, but observed no enrichment of background levels (Figure 3C). Similar results were observed for ectopic expression of WT p53 in HCC1806 cells, with 70-fold and 15-fold enrichment in p53 binding to p21 promoter and *toca1* intron 2, respectively, compared to IgG control (Figure 3D,E). In this system, no increased association of p53 with the proximal promoter of *toca1* was observed (data not shown). In addition, neither of the *toca1* promoter or intron 2 regions were recovered with mutant p53 in ChIP assays performed with MDA-MB-231 cells (data not shown). Together, these results identify a functional binding site for p53 within intron 2 of *toca1*.

Previous studies have identified epigenetic effects of WT p53 activation on its target genes, including effects on histone H3 acetylation at Lys14 (H3K14Ac) and methylation at Lys27 that are associated with transcriptionally active and silent states, respectively [30]. To test this, we extended our ChIP assays on HCC1806 cells transfected with WT p53 plasmid using H3K14Ac antibody, and observed decreased recovery of the *toca1* promoter region with this chromatin mark upon p53 transfection (Figure 3F). Similar results were obtained in DU4475 cells treated with CPT to induce stabilization of endogenous p53, and this resulted in reduced recovery of the *toca1* promoter region with the H3K14Ac mark (Figure 3G). These results are consistent with active transcription of *toca1* under conditions of p53 degradation via MDM2 (murine double minute-2), and the loss of this open chromatin mark upon p53 activation and binding within intron 2 of the *toca1* gene. This may be due to increased recruitment of histone deacetylase complexes (HDACs) that are known to associate with activated p53 and cause repression of some target genes [31]-[34].

### p53 suppresses invadopodia formation and invasion of MTLn3 cells in a Toca-1-dependent manner

**Figure 4:**
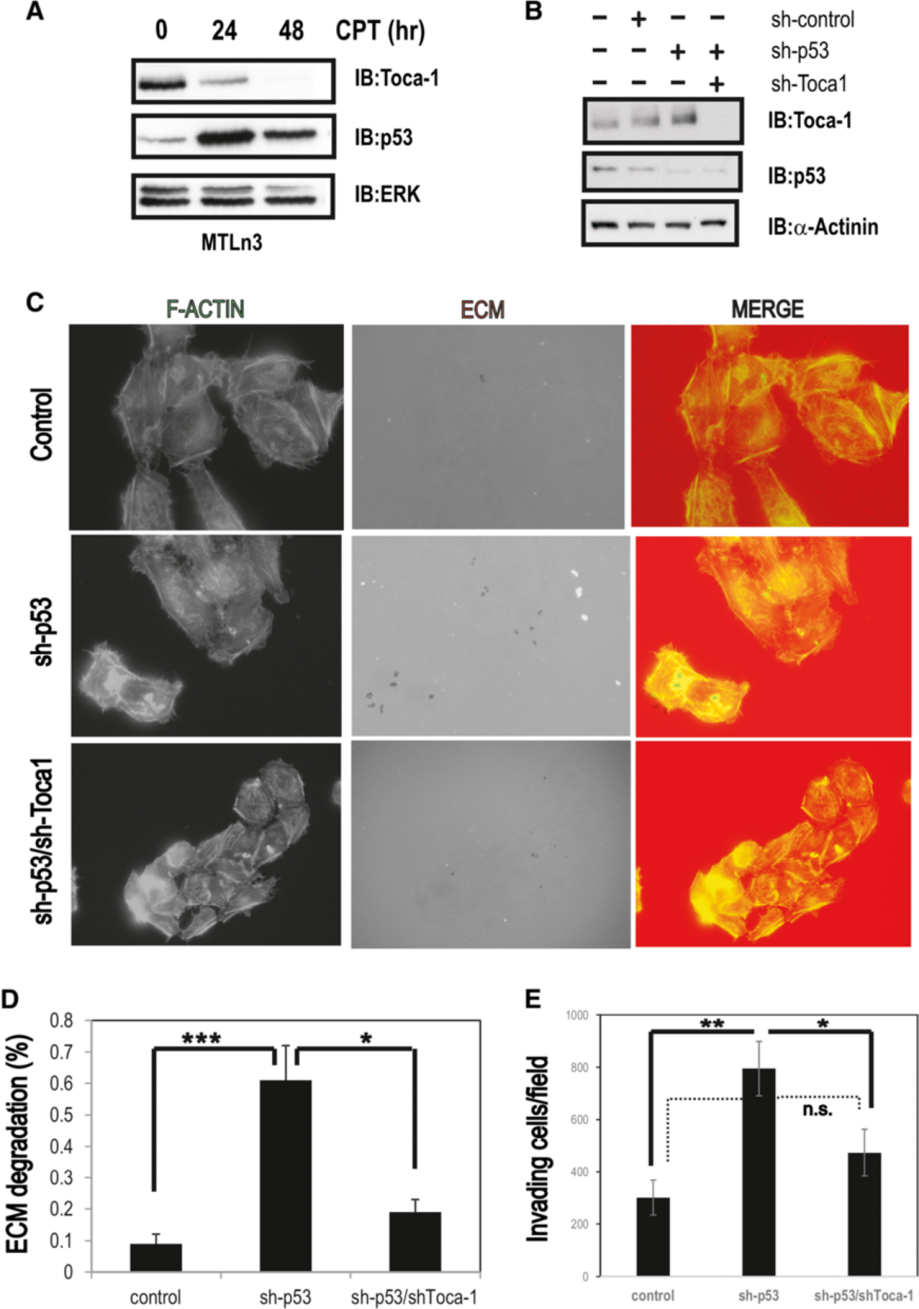
**p53 suppresses Toca-1 expression and invadopodia formation in MTLn3 cells. (A)** MTLn3 cells were treated with CPT (4 μM) for indicated times and analyzed by IB with Toca-1, p53 and ERK antibodies (ERK served as a loading control). **(B)** MTLn3 cells with stable silencing of p53 and Toca-1 alone, or in combination, was tested by IB with Toca-1, and p53 antibodies (α-actinin served as a loading control). **(C)** Representative epiflourescence micrographs for MTLn3 control, sh-p53 and sh-p53/sh-Toca-1 cells plated on TRITC-gelatin (ECM) and stained with phalloidin (F-actin; merge indicates an overlay of both channels). **(D)** Graph depicts the ECM degradation area relative to total cell area for cell lines described above (mean ± s.e.m.; triplicate samples with 12 fields and >40 cells analyzed; ^*^depicts a significant difference based on paired Student’s *t* test (*P* <0.05)). **(E)** Transwell invasion assays for MTLn3 control, sh-p53 and sh-p53/sh-Toca-1 cells. The graph depicts the number of cells per field that had invaded through the ECM to the underside of the filter for each cell line (mean ± s.e.m.; 12 fields were analyzed from triplicate filters for each cell line; ^*^ and ^**^ depict significant differences between the indicated groups (*P* <0.05 and *P* <0.01, respectively) based on paired Student’s *t* test; dashed line indicates no significant difference (n.s.) based on paired Student’s *t* test; results are representative of two independent experiments). CPT, camptothecin; IB, immunoblot; ECM, extracellular matrix; ERK, extracellular signal-regulated kinases; F-actin, filamentous actin; s.e.m., standard error of the mean; Toca-1, transducer of Cdc42-mediated actin assembly; WT, wild-type.

To further relate the p53/Toca-1 axis in the context of breast cancer progression and metastasis, we used rat mammary adenocarcinoma MTLn3 cells that express WT p53 and have been used extensively to model breast cancer metastasis [3],[35],[36]. Further, we recently used this cell model in our study that identified Toca-1 in promoting breast cancer cell invasion and tumor metastasis [11]. First, we confirmed that WT p53 also downregulates rat Toca-1 levels in MTLn3 cells upon treatment with CPT. We observed maximal p53 stabilization at 24 hours, and reduced levels of Toca-1 (Figure 4A). Together with our results in human breast cell lines, this provides further support for a WT p53 pathway that suppresses Toca-1 expression. To test whether p53 regulates the invasive potential of MTLn3 cells, we used retroviral or lentiviral delivery of shRNAs against p53 and Toca-1, respectively, to achieve stable silencing of p53 and Toca-1. We observed efficient depletion of p53 in sh-p53 cells, and this correlated with increased levels of Toca-1 compared to sh-control or parental MTLn3 cells (Figure 4B). These results are consistent with our findings linking WT p53 to Toca-1 levels in human breast cancer cells. In sh-p53/sh-Toca-1 cells, we observed a complete depletion of both Toca-1 and p53 (Figure 4B).

Since p53 was recently shown to repress podosome formation and invasiveness of smooth muscle cells and fibroblasts [18],[19], we tested the effects of p53 silencing on invadopodia formation in MTLn3 cells. MTLn3 control and sh-p53 cells were plated on coverslips coated with fluorescent ECM (thin gelatin) for 16 hours prior to staining of F-actin. Epifluorescence micrographs were obtained to visualize ECM degradation spots relative to F-actin dots that mark invadopodia. As expected, control MTLn3 cells degraded ECM, but the amount of degradation spots and F-actin dots was enhanced with p53 silencing (Figure 4C). Quantification of these results revealed a 2.5-fold increase in ECM digestion area per cell with p53 silencing compared to control MTLn3 cells (Figure 4D). A parallel analysis of p53/Toca-1 double KD cells revealed a partial rescue of the effects of p53 silencing (Figure 4C,D). These results are consistent with Toca-1 acting as a positive regulator of invadopodia [11], and provide evidence that p53 mutation status likely contributes to the ability of cancer cells to form invadopodia through altered expression of invadopodia regulatory proteins.

To test whether p53 also regulates the ability of MTLn3 cells to invade through ECM, MTLn3 control, sh-p53 and sh-p53/sh-Toca-1 cells were plated in transwell chambers overlayed with Matrigel, and invasion toward the lower chamber was measured after 24 hours. We found that silencing of p53 significantly increased the number of invading cells compared to control MTLn3 cells (Figure 4E). However, dual silencing of Toca-1 and p53 led to a significant reduction in cell invasion compared to sh-p53 cells, which returned to near baseline levels in control MTLn3 cells (Figure 4E). Taken together, these results provide novel evidence for p53 suppression of invadopodia in cancer cells, and identify Toca-1 as a key participant in ECM degradation and cell invasion upon loss of p53.

### Silencing p53 enhances MTLn3 tumor growth and metastasis and is partially dependent on Toca-1

**Figure 5:**
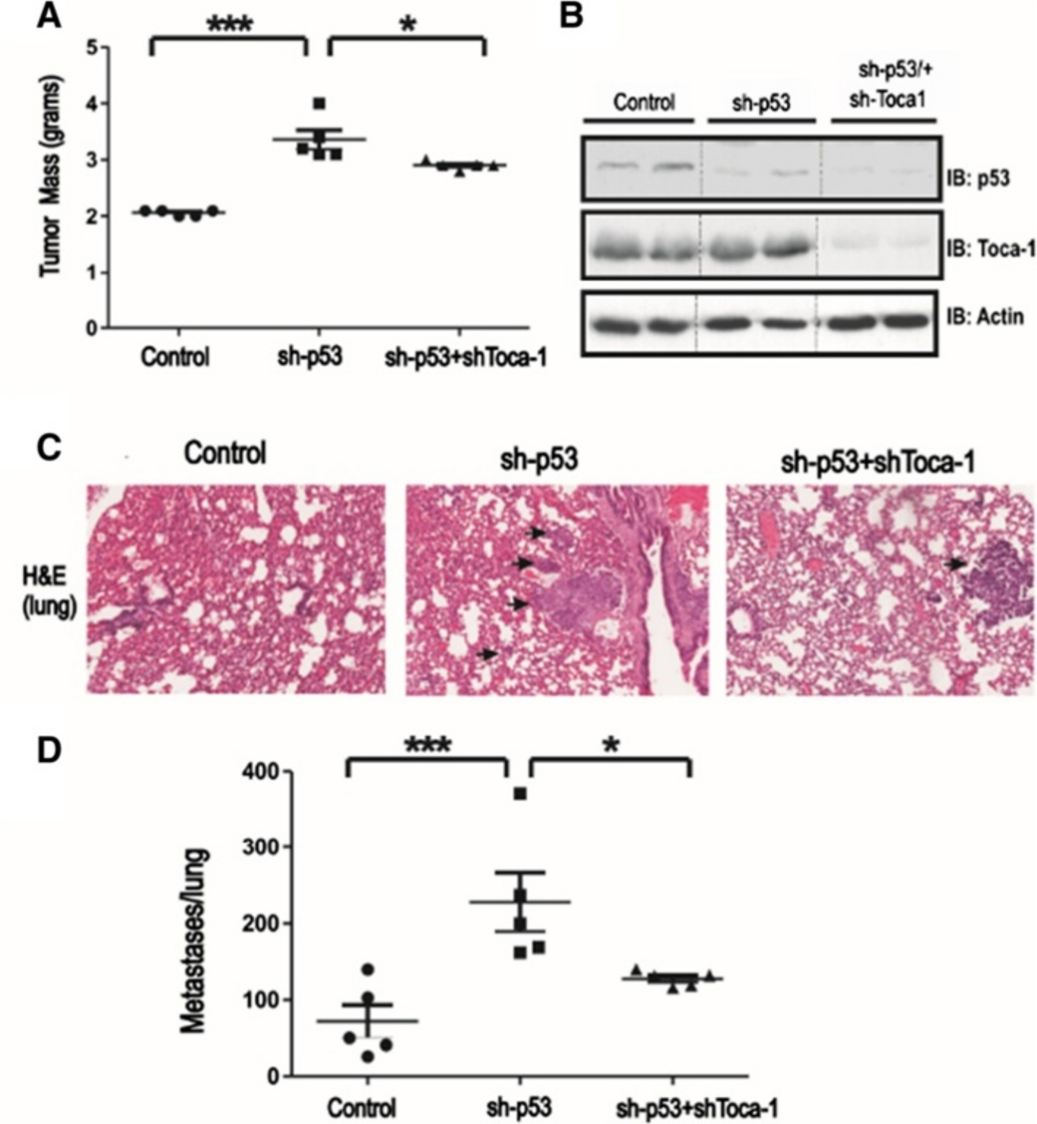
**p53 silencing enhances MTLn3 tumor progression and lung metastasis and partial rescue with Toca-1 silencing.** Mammary orthotopic xenograft assays using MTLn3 control, sh-p53 and sh-p53/sh-Toca-1 cells were analyzed at 5 weeks post injection of Rag2^-/-^IL2Rγc^-/-^ mice. **(A)** Graph depicts primary tumor mass for each mouse injected with the indicated cell line (mean ± SD; n = 5 mice/group; significant differences between groups based on paired Student’s *t* test are indicated by ^*^P <0.05 or ^***^P <0.001). **(B)** Levels of Toca-1 and p53 in the tumor homogenates were analyzed by IB (β-actin served as a loading control; dashed lines indicating removal of additional lanes). **(C)** Representative H&E-stained sections of lungs from mice harboring mammary tumors for the indicated cell lines are shown (arrows depict metastatic nodules). **(D)** Graph depicts the number of metastases per lung for each mouse injected with the indicated cell line (mean ± SD; n = 5 mice/group; significant differences between groups based on paired Student’s *t* test are indicated by ^*^P <0.05 or ^***^P <0.001). H&E, hematoxylin and eosin; IB, immunoblot; SD, standard deviation; Toca-1, transducer of Cdc42-mediated actin assembly.

To investigate contributions of the p53/Toca-1 axis to breast cancer progression and mestastasis, we performed mammary orthotopic xenograft assays using MTLn3 control, sh-p53 and sh-p53/sh-Toca-1 cells in Rag2^−/−^:IL2Rγc^−/−^ mice. After 5 weeks, mammary tumors were harvested, and we observed a significant increase in tumor mass with silencing of p53 compared to control (Figure 5A). However, a significant reduction in tumor mass was observed upon combined silencing of Toca-1 and p53. These differences in tumor growth were not due to inherent differences in cell viability or growth rates *in vitro* (Figure S3 in Additional file 3). Tumor homogenates were subjected to IB with p53 and Toca-1 antisera, which demonstrated the continued silencing of these proteins during the tumor study (Figure 5B). Since this model results in metastasis to the lungs of these mice [11],[22], we analyzed lung tissue sections for presence of metastatic nodules. Interestingly, we observed increased lung metastases with p53 silencing in MTLn3 tumor-bearing mice (Figure 5C,D). However, combined silencing of Toca-1 and p53 led to a partial rescue of the lung metastasis phenotype compared to control MTLn3 (Figure 5C,D). To address whether these defects might relate to altered tumor mass between groups, we tested for a correlation in our MTLn3 xenograft model. We observed a positive correlation between tumor mass and the numbers of lung metastases that was significant, but with a poor correlation co-efficient (R2 = 0.377; Figure S4 in Additional file 4). However, this is unlikely to explain the effects of combined p53 and Toca-1 silencing since differences in tumor mass were quite modest compared to sh-p53 tumors (Figure 5A). Together, these results provide evidence for suppression of breast tumor progression and metastasis by WT p53, and that Toca-1 contributes to the increased metastasis with p53 loss-of-function.

### Toca-1 is highly expressed in human breast cancer subtypes with frequent p53 mutations and a potential marker of poor prognosis

**Figure 6:**
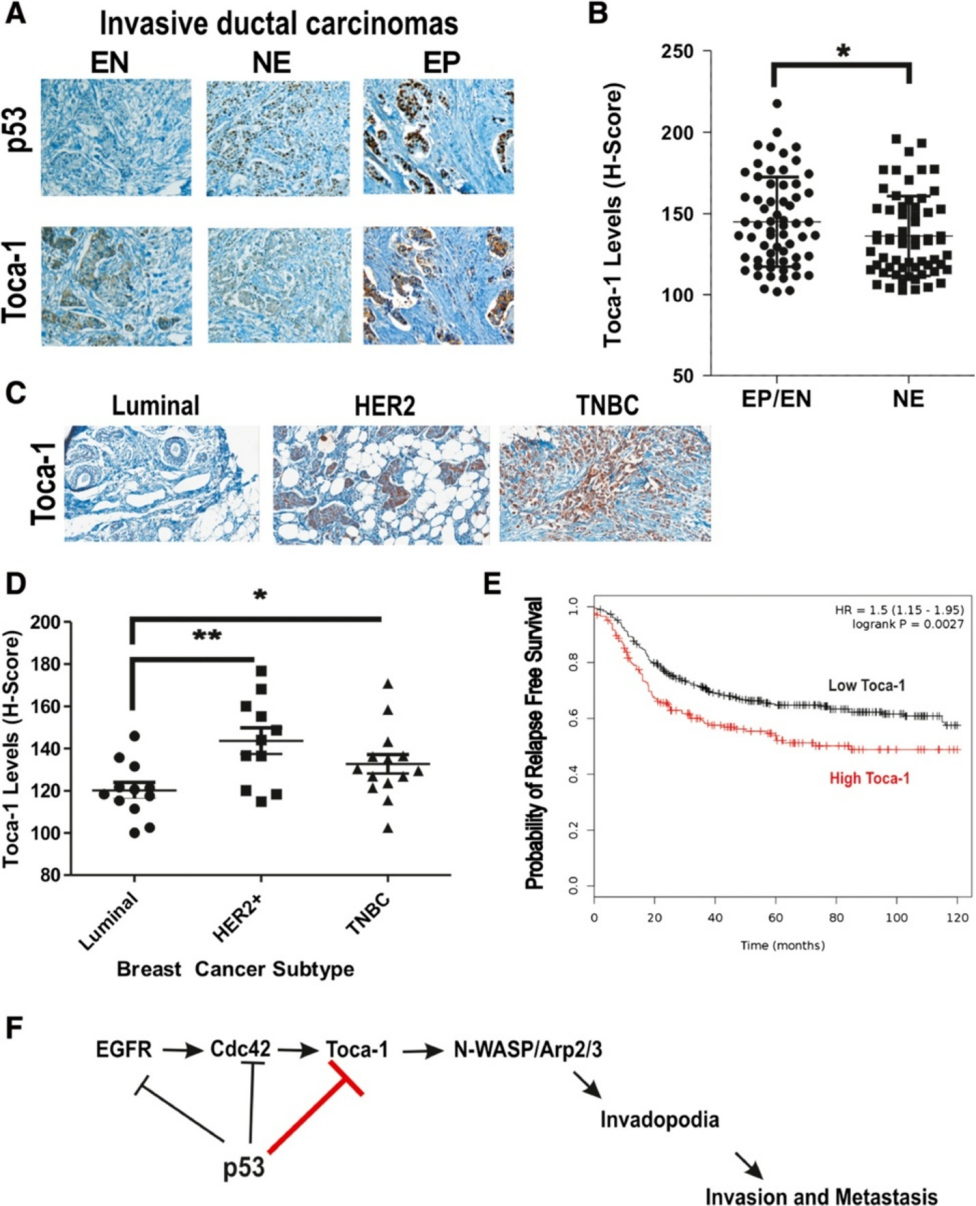
**Toca-1 is highly expressed breast cancer subtypes with frequent p53 mutations and is associated with poor prognosis. (A)** Representative micrographs showing the range of expression of Toca-1 and p53 in human invasive ductal carcinomas based on IHC staining. **(B)** Graph depicts quantification of Toca-1 levels using H-score analysis according to p53 IHC staining patterns (EP/EN, extreme positive/extreme negative; NE, non-extreme or intermediate levels) in 63 human ductal carcinomas (^*^indicates a significant difference between groups based on paired Student’s *t* test, *P* <0.05). **(C)** Representative micrographs showing results of Toca-1 IHC staining in human breast cancer subtypes, including luminal, HER2 and TNBCs. **(D)** Graph depicts quantification of Toca-1 IHC staining using H-score analysis, and grouped by molecular subtypes (luminal, HER2 and TNBC; significant differences between groups based on paired Student’s *t* test are indicated by ^*^P <0.05 or ^**^P <0.01). **(E)** Kaplan-Meier Plotter analysis of basal breast cancer patients with high or low Toca-1 transcript levels according to relapse-free survival (n = 581; HR = 1.5, logrank *P* <0.01). **(F)** A schematic model depicting known targets of WT p53 (EGFR, Cdc42, Toca-1) that upon p53 loss or mutation, promote formation of invadopodia, invasion through ECM, and tumor metastasis. ECM, extracellular matrix; EGFR, epidermal growth factor receptor; IHC, immunohistochemistry; HER2, human epidermal growth factor receptor 2; HR, hazard ratio; TNBCs, triple-negative breast cancers; Toca-1, transducer of Cdc42-mediated actin assembly.

Having established a novel link between Toca-1 and p53, we aimed at expanding our analysis to human primary breast cancers. Human breast tumor tissue microarrays with known ER, PR, and HER2 status were stained with Toca-1 and p53 antibodies by IHC. As expected, the levels of p53 and Toca-1 varied greatly within tumor cores (Figure 6A). In the case of p53, we grouped these cases as uniformly negative (extreme negative, EN), intermediate or variable levels (non-extreme, NE), and uniformly positive (extreme positive, EP) based on a recent study showing that the majority of extreme p53 cases (EP/EN) correspond to p53 mutations [24]. Interestingly, this study also showed that p53 EP/EN patients had worse overall survival. To relate Toca-1 levels to p53 IHC staining categories, we determined H-scores for Toca-1 in 63 cases with p53 status of EP/EN, and 63 cases with NE status (Figure 6B). Interestingly, there was significantly higher median expression of Toca-1 in p53 EP/EN category compared to NE (Figure 6B). We also extended the analysis of Toca-1 levels to the major molecular subtypes, including luminal, HER2 and TNBC. Consistent with the increased frequency of p53 mutations in HER2 and TNBC subtypes [37], and our profiling of Toca-1 in breast cancer cell lines [11], we observed significantly increased Toca-1 expression levels in these subtypes compared to luminal tumors (Figure 6C,D). This is also consistent with increased frequency of extreme p53 expression by IHC (EP/EN) in HER2 and TNBC compared to luminal [24], and their relative risk of metastasis [38],[39]. Thus, higher Toca-1 levels may be due to loss of WT p53 pathways in HER2 or TNBC tumors. To test whether high Toca-1 levels correlated with clinical outcomes, we used the Kaplan-Meier Plotter analysis tool [40] to analyze Toca-1 transcript levels in breast cancer microarray datasets. Interestingly, we observed a significant association between high Toca-1 transcript levels and increased risk of relapse in basal breast cancer patients (Figure 6E; hazard ratio = 1.43, *P* <0.05). Since the majority of TNBCs have been classified as basal breast cancers [41],[42], the reduced relapse-free survival in patients with high Toca-1 levels reported here is consistent with our functional studies implicating Toca-1 in promoting breast cancer cell invasion and tumor metastasis downstream of EGFR and Cdc42. Thus, we postulate that in normal cells, or cancer cells with WT p53 function, this EGFR/Cdc42/Toca-1 pathway can be suppressed at multiple points by WT p53 (Figure 6F).

## Discussion

The current study describes a novel mechanism to limit expression of the breast cancer invasion and metastasis-promoting protein Toca-1, by the tumor suppressor p53. Breast cancers with loss of p53, or frequent p53 mutations (HER2, TNBC), have high rates of metastasis [38],[39]. This is due to activation of EGFR/HER2 and Src signaling pathways in these cancers, leading to formation of invadopodia, and increased rates of metastasis [43]-[46]. We previously reported that Toca-1 promotes breast cancer cell invasion and tumor metastasis [11], and were interested in defining how Toca-1 expression is regulated. Here, we report that Toca-1 expression in normal breast epithelial cells and breast cancers is significantly associated with p53 status. Activation of WT p53 led to decreased expression of Toca-1, which involves the binding of p53 within intron 2 and decreased open chromatin marks within the promoter region of the *toca1* gene. Using MTLn3 breast cancer cells expressing WT p53, we provide evidence that p53 can partially suppress invadopodia, cell invasion, and tumor metastasis. The combined silencing of p53 and Toca-1 significantly reduced the phenotypes observed with p53 silencing alone. Lastly, we correlated high Toca-1 levels with HER2 and TNBC subtypes that frequently harbor mutations in tumor suppressor p53 [47], and also with extreme IHC staining (negative or positive) of p53 in our invasive ductal carcinoma tissue microarrays. Based on these observations, we propose a model whereby WT p53 inhibits the EGFR/Cdc42/Toca-1 signaling axis in normal cells, and some breast cancers, to suppress the invasive phenotype of these cells (Figure 6F).

We postulate that p53 suppresses Toca-1 in some cancer cells to limit their invasive potential. The loss of WT p53 via frequent mutations observed in HER2 and TNBCs, likely explains the high levels of Toca-1 in these tumors and cell lines. However, the mutant p53 is more stable than WT p53, and accumulates in the nucleus and interacts with many oncoproteins resulting in gain-of-function effects [48]. In terms of Toca-1 expression, we observed no effect of either overexpression of mutant p53 or silencing the endogenous mutant p53 in HER2 or TNBC cell lines. Together with evidence that Toca-1 is repressed by WT p53, the higher levels of Toca-1 we observed in HER2 and TNBC tumors and cell lines, is likely explained by loss of WT p53-mediated gene repression. Our results also identify a p53 binding site within intron 2 of *toca1*, and reduced open chromatin marks within the promoter region of *toca1*. This mechanism is consistent with the repression of Survivin by p53, which involves recruitment of HDACs to the Survivin promoter region leading to chromatin condensation [32]-[34]. Thus, a similar mechanism may be involved in the repression of Toca-1 following p53 activation. Other known p53 target genes with intronic p53 response elements include GADD45 [49],[50], and pregnancy-associated plasma protein A [23]. Future studies will be required to fully define the epigenetic marks and p53-associated factors involved in this mechanism to limit Toca-1 expression.

Although the best characterized tumor suppressor role for p53 involves the DNA damage response, there is a growing appreciation for its role as a cell invasion suppressor [17]. A number of p53 target genes have been implicated in cancer cell invasion and metastasis [51]-[54]. For example, a recent study showed that mammary-specific inactivation of E-cadherin and p53 leads to pleomorphic invasive lobular carcinoma in mice [52]. In stromal fibroblasts, p53 has been shown to attenuate cancer cell migration and invasion by repressing SDF-1/CXCL12 expression [53]. WT p53 also regulates MET receptor and has been shown to control cell motility and invasion in ovarian cancer [54]. WT p53 also inhibits EGFR expression, which is a key pathway in TNBC metastasis [15]. Other targets of p53 are caldesmon, a negative regulator of Src-induced podosome formation and ECM degradation [19], and microRNAs (miR-143/miR-145) that target key pathways leading to podosome formation [18]. Although the latter studies suggest that WT p53 will suppress invadopodia, due to similarities in pathways and components for podosomes, this has not been previously reported. Here, we used the MTLn3 model with stable silencing of p53 to show that cell invasion and tumor metastasis remain under some level of control by p53 in this breast cancer model. Furthermore, the loss of WT p53-mediated suppression of Toca-1 contributes the increased cell invasion and tumor metastasis upon p53 silencing in this breast cancer model. Importantly, increased expression of Toca-1 in human breast tumors from HER2 and TNBC subtypes with frequent p53 mutations, further supports the relevance of this p53/Toca-1 signaling axis in cancer progression. Certainly, the increased risk of relapse in basal breast cancer patients with high Toca-1 transcript levels would fit with our evidence for Toca-1 promoting metastasis, but should be investigated further at the protein level.

## Conclusions

In conclusion, our results demonstrate that accumulation of WT p53 leads to repression of Toca-1, and loss of this regulatory axis likely contributes to upregulation of Toca-1 in highly metastatic cancers (Figure 6F). Based on our study, we identify Toca-1 as a pro-invasion/pro-metastasis protein that is downregulated by WT p53 in both rat and human breast cancer models. This may reflect the fact that Toca-1 is capable of scaffolding and activating numerous regulators of actin assembly at cell protrusions that facilitate motility and invasion. Thus, limiting Toca-1 levels could reduce a number of pathways that attempt to activate these actin-regulatory proteins in normal cell and cancer cells.

## Authors’ information

HC is a breast cancer biologist that trained as a post-doctoral fellow with both DG (2006 to 2010) and AWBC (2010 to 2014). HC is now an Assistant Professor in the Centre for Genetic Diseases and Molecular Medicine, Central University of Punjab, Bathinda, India. AWBC is a cancer biologist specializing in molecular mechanisms of cancer metastasis, and an Associate Professor at Queen’s University (2002 to 14). AWBC received the Canadian Cancer Society Young Investigator Award (2011), and the Canadian Institutes for Health Research New Investigator Award (2004 to 2009).

## Additional files

## Electronic supplementary material

Additional file 1: Figure S1.: Toca-1 transcript level profiling in breast cancer cell lines according to p53 mutation status. (PDF 150 KB)

Additional file 2: Figure S2.: Toca-1 downregulation by p53 in MCF10A breast epithelial cells. (PDF 188 KB)

Additional file 3: Figure S3.: Silencing of p53 and Toca-1 does not affect viability or growth of MTLn3 cells. (PDF 78 KB)

Additional file 4: Figure S4.: Correlation between lung metastases and primary tumor mass in mammary orthotopic MTLn3 xenograft assays. (PDF 99 KB)

Below are the links to the authors’ original submitted files for images.

Authors’ original file for figure 1

Authors’ original file for figure 2

Authors’ original file for figure 3

Authors’ original file for figure 4

Authors’ original file for figure 5

Authors’ original file for figure 6

## Abbreviations

BSA: bovine serum albumin
ChIP: chromatin immunoprecipitation
CIP4: Cdc42-interacting protein-4
CPT: camptothecin
DMEM: Dulbecco’s modified Eagle’s medium
ECM: extracellular matrix
EGFR: epidermal growth factor receptor
EMT: epithelial-mesenchymal transition
EN: extreme negative
EP: extreme positive
ER: estrogen receptor
F-actin: filamentous actin
F-BAR: Fer/CIP4 homology-Bin/Amphiphysin/Rvs
FBS: fetal bovine serum
GAPDH: glyceraldehyde-3-phosphate dehydrogenase
H&E: hematoxylin and eosin
H3K14Ac: histone H3 K14 acetylated
HDACs: histone deacetylase complexes
HER2: human epidermal growth factor receptor 2
HR1: PKN homology region 1
HRP: horseradish peroxidase
IB: immunoblot
IgG: immunoglobulin G
IHC: immunohistochemistry
KN: knockdown
miRNAs: microRNAs
NE: non-extreme
PR: progesterone receptor
qPCR: quantitative polymerase chain reaction
RT: reverse transcription
shRNA: short hairpin RNA
siRNA: small interfering RNA
TNBC: triple-negative breast cancer
Toca-1: transducer of Cdc42-mediated actin assembly
WT: wild-type
