# Supplementary material for: Toca-1 is suppressed by p53 to limit breast cancer cell invasion and tumor metastasis
Source: Breast Cancer Res. 2014 Dec 30;16:3413. doi: 10.1186/s13058-014-0503-x (PMC4332744; doi:10.1186/s13058-014-0503-x)
Supplement: Supplementary file 1 — Additional file 1: Figure S1.: Toca-1 transcript level profiling in breast cancer cell lines according to p53 mutation status. (PDF 150 KB) [file 13058_2014_503_MOESM1_ESM.pdf]

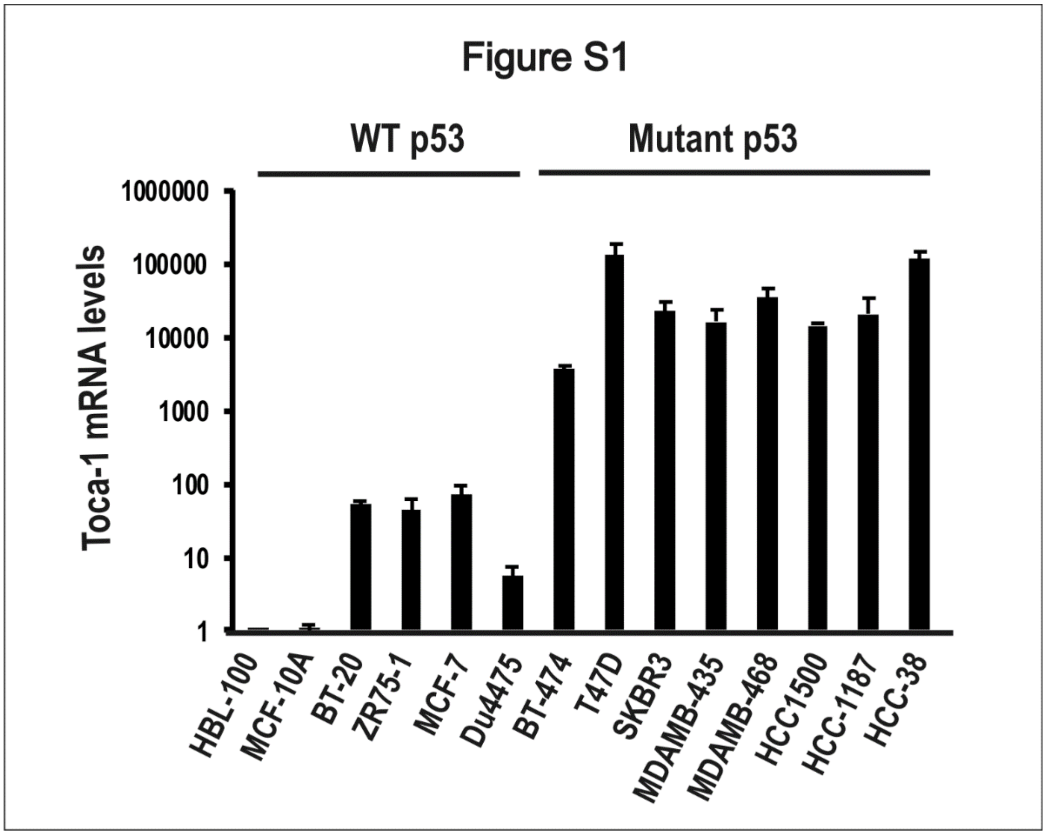

Figure S1. Toca-1 transcript level profiling in breast cancer cell lines according to p53 mutation status. Toca-1 mRNA levels were measured for the same panel of cell lines by qRT-PCR ($2^{-\Delta\Delta CT}$ values for Toca-1 were normalized to GAPDH for each cell line, and graph depicts transcript levels relative to HBL-100 cells (mean ± SD; triplicate samples).
