# Supplementary material for: Toca-1 is suppressed by p53 to limit breast cancer cell invasion and tumor metastasis
Source: Breast Cancer Res. 2014 Dec 30;16:3413. doi: 10.1186/s13058-014-0503-x (PMC4332744; doi:10.1186/s13058-014-0503-x)
Supplement: Supplementary file 2 — Additional file 2: Figure S2.: Toca-1 downregulation by p53 in MCF10A breast epithelial cells. (PDF 188 KB) [file 13058_2014_503_MOESM2_ESM.pdf]

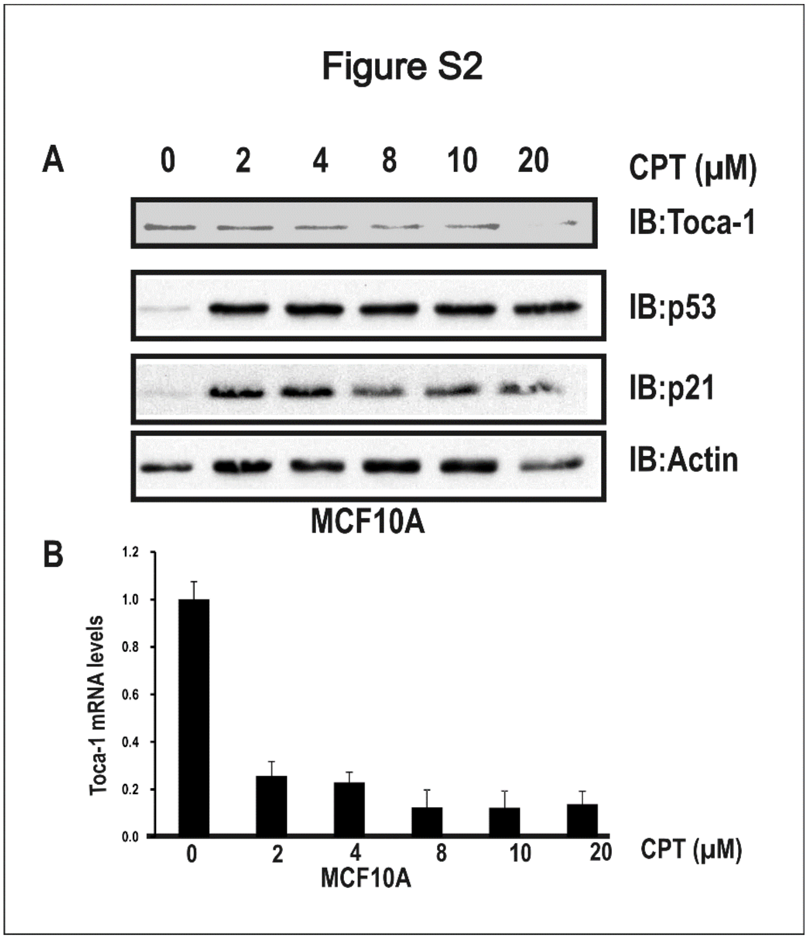

Figure S2. Toca-1 downregulation by p53 in MCF10A breast epithelial cells **(A)** MCF10A were treated with the indicated doses of CPT for 24 hours. Lysates were subjected to IB for Toca-1, p53, p21, and β-actin. **(B)** RNA was isolated from MCF10A cells treated as above for qRT-PCR analysis of Toca-1 mRNA levels ($2^{-\Delta\Delta CT}$ values for Toca-1 were normalized to GAPDH for each concentration of the drug, and graph depicts transcript levels relative to DMSO treated cells (mean ± SD; triplicate samples)).
