# Supplementary material for: Toca-1 is suppressed by p53 to limit breast cancer cell invasion and tumor metastasis
Source: Breast Cancer Res. 2014 Dec 30;16:3413. doi: 10.1186/s13058-014-0503-x (PMC4332744; doi:10.1186/s13058-014-0503-x)
Supplement: Supplementary file 3 — Additional file 3: Figure S3.: Silencing of p53 and Toca-1 does not affect viability or growth of MTLn3 cells. (PDF 78 KB) [file 13058_2014_503_MOESM3_ESM.pdf]

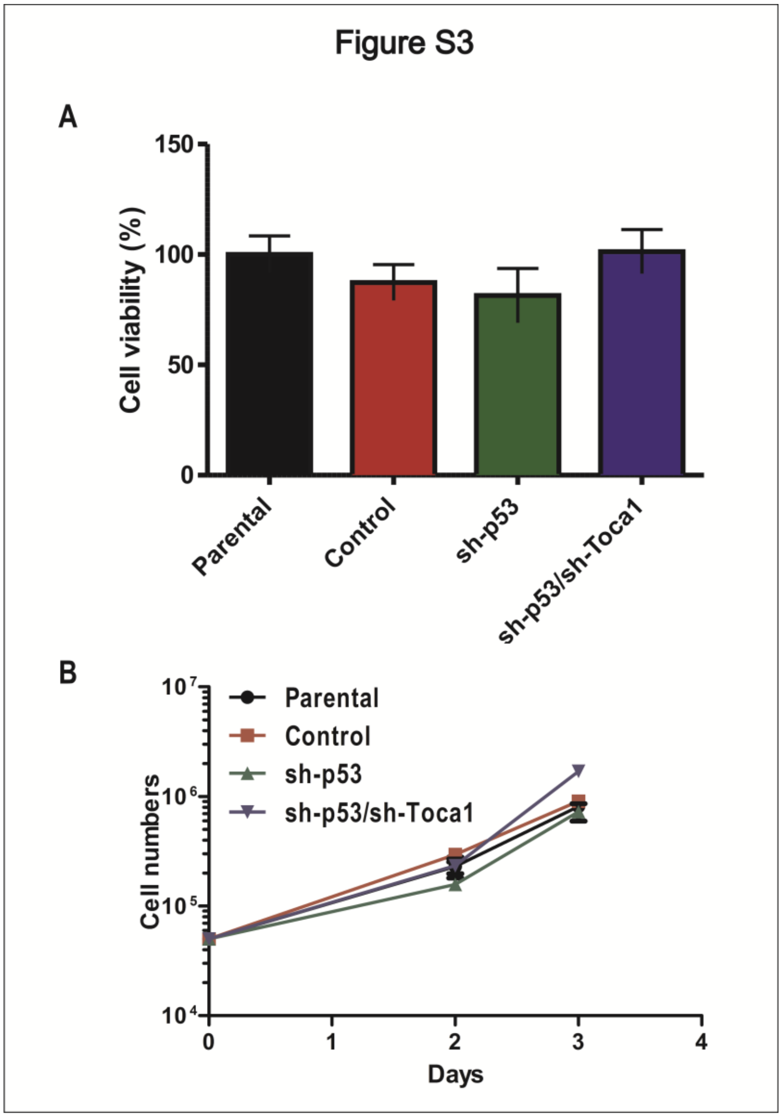

Figure S3. Silencing of p53 and Toca-1 does not affect viability or growth of MTLn3 cells **(A)** Cell viability was measured using AlamarBlue® assay for MTLn3 parental cells, shRNA control cells, sh-p53 cells and sh-p53/sh-Toca-1 cells as described in Methods. Graph depicts mean absorbance values (± SD) for MTLn3 derivates relative to parental cells. **(B)** Cell growth rates of MTLn3 parental cells, shRNA control cells, sh-p53 cells and sh-p53/sh-Toca-1 cells were measured at days 2 and 3 post seeding. No significant differences in cell viability or cell growth were observed between cell lines.
