# Supplementary material for: Toca-1 is suppressed by p53 to limit breast cancer cell invasion and tumor metastasis
Source: Breast Cancer Res. 2014 Dec 30;16:3413. doi: 10.1186/s13058-014-0503-x (PMC4332744; doi:10.1186/s13058-014-0503-x)
Supplement: Supplementary file 4 — Additional file 4: Figure S4.: Correlation between lung metastases and primary tumor mass in mammary orthotopic MTLn3 xenograft assays. (PDF 99 KB) [file 13058_2014_503_MOESM4_ESM.pdf]

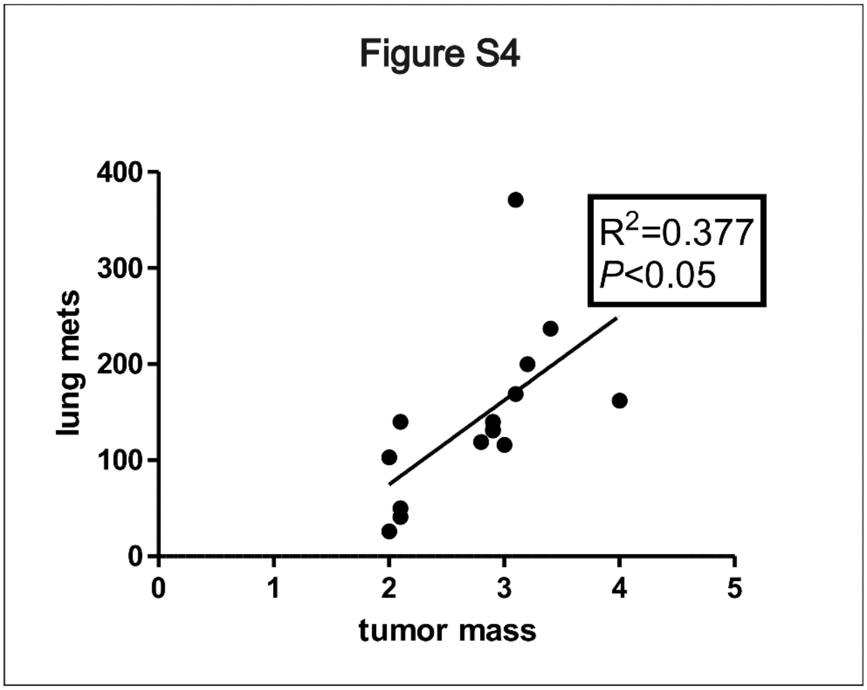

Figure S4. Correlation between lung metastases and primary tumor mass in mammary orthotopic MTLn3 xenograft assays. Graph depicts a comparison of primary tumor mass and number of lung metastases in MTLn3 control, sh-p53 and sh-p53/sh-Toca1 cell lines. Although a positive correlation was observed (P<0.05), the correlation co-efficient was only 0.377.
