# Supplementary figures and images for: Toca-1 is suppressed by p53 to limit breast cancer cell invasion and tumor metastasis
Source: Breast Cancer Res. 2014 Dec 30;16:3413. doi: 10.1186/s13058-014-0503-x (PMC4332744; doi:10.1186/s13058-014-0503-x)

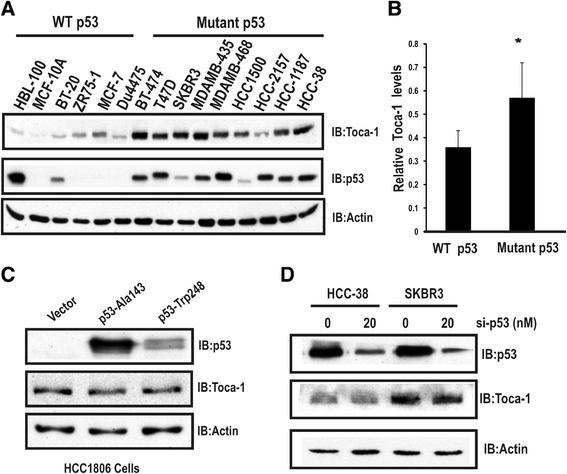

Supplement: Supplementary file 5 — Authors’ original file for figure 1 [file 13058_2014_503_MOESM5_ESM.gif]

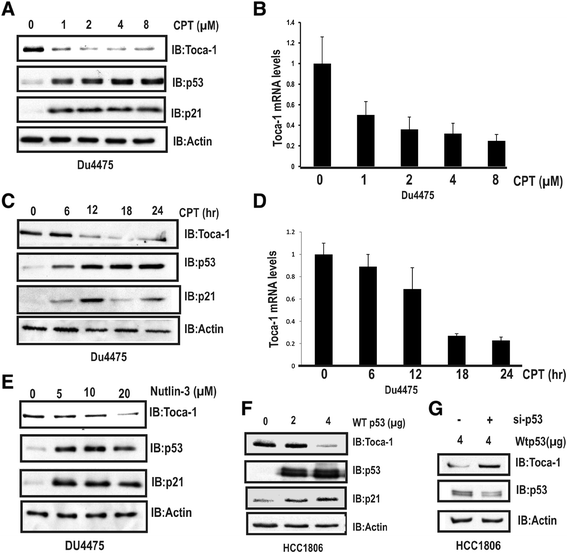

Supplement: Supplementary file 6 — Authors’ original file for figure 2 [file 13058_2014_503_MOESM6_ESM.gif]

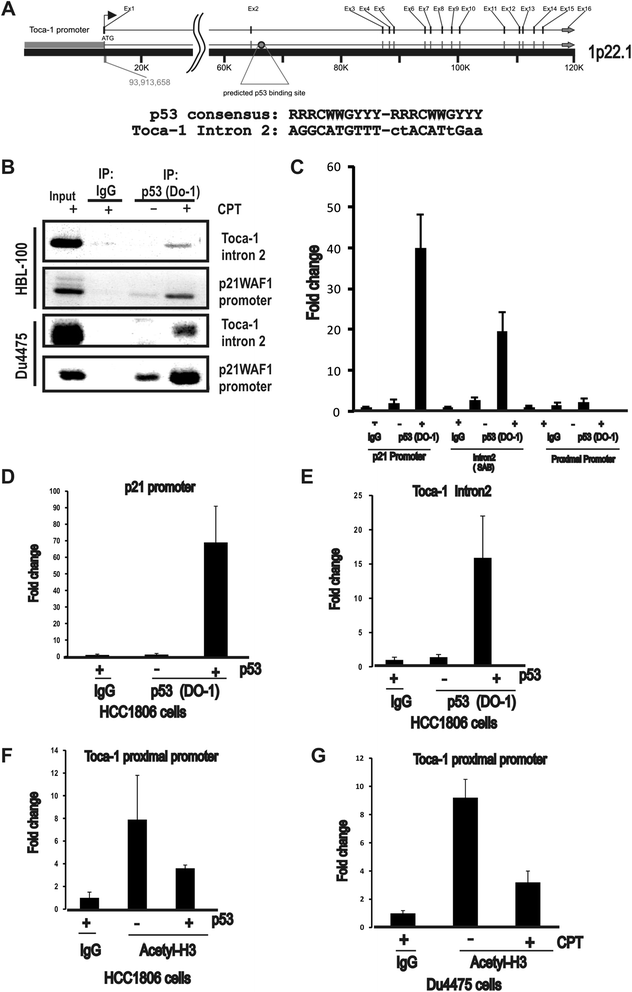

Supplement: Supplementary file 7 — Authors’ original file for figure 3 [file 13058_2014_503_MOESM7_ESM.gif]

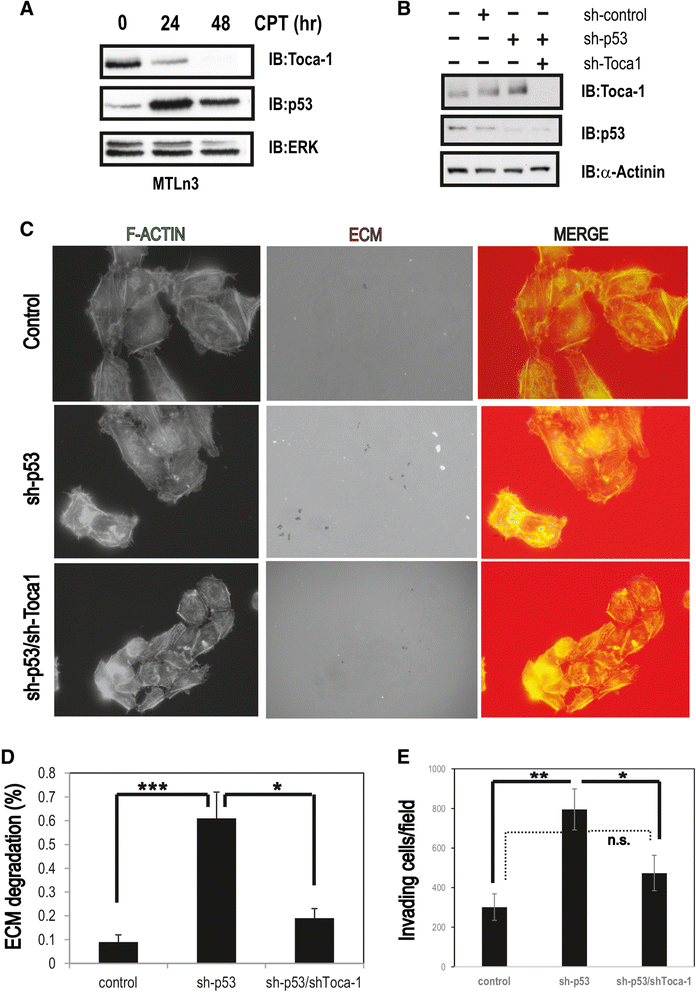

Supplement: Supplementary file 8 — Authors’ original file for figure 4 [file 13058_2014_503_MOESM8_ESM.gif]

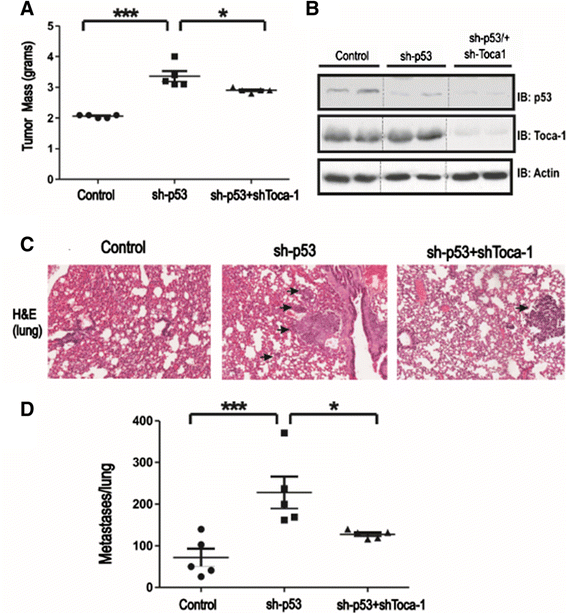

Supplement: Supplementary file 9 — Authors’ original file for figure 5 [file 13058_2014_503_MOESM9_ESM.gif]

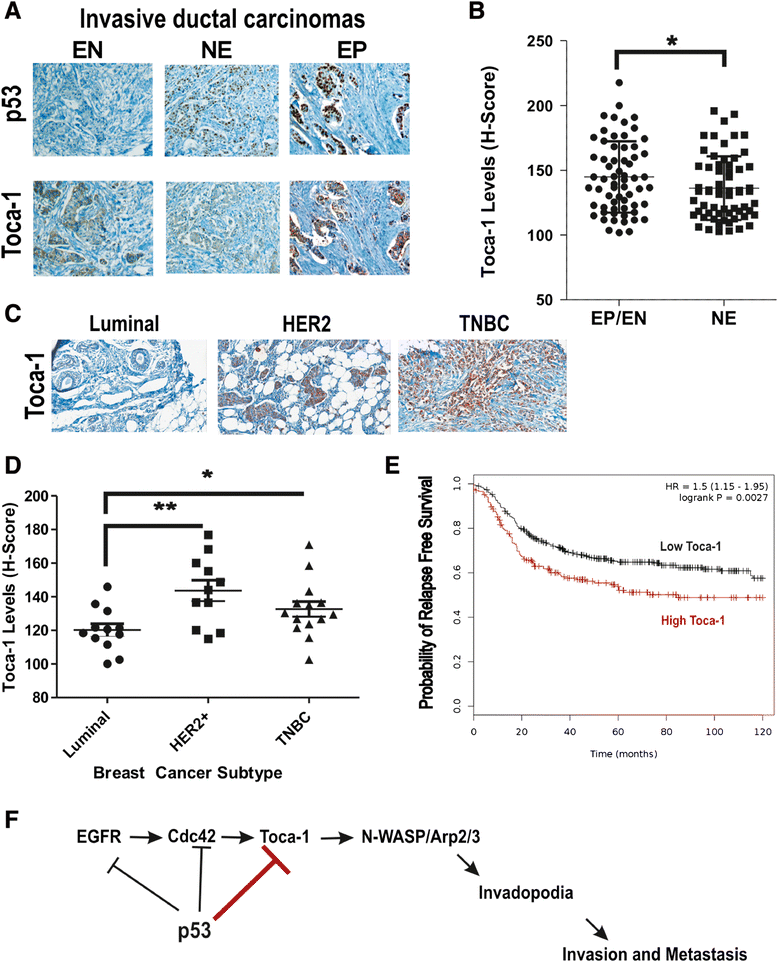

Supplement: Supplementary file 10 — Authors’ original file for figure 6 [file 13058_2014_503_MOESM10_ESM.gif]
